# Predictors of poor prognosis in ANCA-associated vasculitis (AAV): a single-center prospective study of inpatients in China

**DOI:** 10.1007/s10238-022-00915-z

**Published:** 2022-10-16

**Authors:** Ronglin Gao, Zhenzhen Wu, Xianghuai Xu, Jincheng Pu, Shengnan Pan, Youwei Zhang, Shuqi Zhuang, Lufei Yang, Yuanyuan Liang, Jiamin Song, Jianping Tang, Xuan Wang

**Affiliations:** 1 Department of Rheumatology and Immunology, Tongji Hospital, School of Medicine, Tongji University, No. 389 Xincun Road, Shanghai, 200065 China; 2 Department of Pulmonary and Critical Care Medicine, Tongji Hospital, School of Medicine, Tongji University, No. 389 Xincun Road, Shanghai, 200065 China

**Keywords:** ANCA-associated vasculitis, Mortality, Renal replace therapy, Tumor, Risk factors/ predictors

## Abstract

To identify potential predictors by assessing adverse outcomes in ANCA-associated vasculitis (AAV) patients. Eighty-nine untreated AAV patients were followed up to January 31, 2022, death, or loss of follow-up. Clinical characteristics, laboratory tests, treatment, and progress were collected, and disease activity was evaluated via Birmingham Vasculitis Activity Score (BVAS). We determined risk factors of high-risk events, defined as developing tumors, renal replacement therapy (RRT), and death. Patients and renal survivals were computed by the Kaplan–Meier curve analysis. Cox regression analysis was performed for assessing variables for predicting death. During 267 person-years follow-up, 46 patients occurred high-risk events, including 20 patients receiving RRT, 12 patients developing tumors, and 29 patients who died mostly from organ failure and infection. Decreased estimated glomerular filtration rate (eGFR) (*P* < 0.001) and complement 3 levels (*P* = 0.019) were associated with high-risk events. Patients with lower serum potassium tended to develop tumors (*P* = 0.033); with higher BVAS (HR = 1.290, 95%CI 1.075–1.549, *P* = 0.006) and lower eGFR (HR = 0.782, 95%CI 0.680–0.901, *P* = 0.001) were more likely to undergo RRT. Patients with cardio and renal involvement exhibited a lower frequency of renal survival and all-cause mortality. Through multivariate COX analysis, age (HR = 1.016, 95%CI 1.016–1.105, *P* = 0.006) and eGFR (HR = 0.982, 95%CI 0.968–0.997, *P* = 0.018) predicted death in AAV, separately. The BVAS and eGFR could be a great prognosticator for RRT, while age and eGFR can independently predict the death. Serum potassium level and immunoglobulins should be focused on their predictor value in development of cancer and renal outcomes in AAV patients.

**Supplementary Information:**

The online version contains supplementary material available at 10.1007/s10238-022-00915-z.

## Introduction

ANCA-associated vasculitis (AAV) is an autoimmune disease with necrotizing inflammation of small vessels as the main manifestation, and the most prominent feature is ANCA positive [1]. Microscopic polyangiitis (MPA) and myeloperoxidase (MPO)-ANCA have a significant predominance in Chinese AAV patients [2]. Research on the etiology of AAV is still unclear. Environmental, genetic, infectious, and immune abnormalities may all be involved in the development of vasculitis [1]. B cells, which produce ANCA-activating neutrophils, are considered to be key players in the pathogenesis of AAV disease [3]. Activated neutrophils eventually lead to vascular endothelial and tissue damage in AAV through the release of neutrophil extracellular traps (NETS), cytotoxic substances, and activation of complement replacement pathways. The continuous stimulation of B cells by antigen and B cell-activating factor (BAFF) released by neutrophils also further promotes the autoimmune response of B cells [4]. It causes irreversible organ damage clinically, especially the kidney which is one of the most commonly involved sites, and finally is associated with poor prognosis or increased mortality [5]. Untreated kidney involvement rapidly progresses to end-stage renal disease (ESRD) and in some cases requires dialysis treatment or kidney transplantation [6].

As we know, the mortality rate of AAV is high due to the absence of immunosuppressive drugs in the early [7]; the advent of hormonal combined with cyclophosphamide (CYC) therapy greatly improves patient’s survival and provides some degree of disease remission [8]. But the long-term prognosis of AAV still remains unsatisfactory. It is associated with some adverse side effects of drugs and is a long-term chronic burden of patients with AAV. According to research, the causes of death in AAV can vary: early in the disease diagnosis, the main causes include active vasculitis and infection, but later on, especially chronic complications such as malignancy and cardiovascular diseases (CVD) emerge [9]. Interestingly, related studies have reported a dose–response relationship between increased risk of malignancy after AAV diagnosis and exposure to CYC [10], however, controversy remains regarding whether there is a common pathogenic pathway between the disease itself and malignancy [11], and on potential cancer risk factors in AAV which is a lack of relevant data. Because of the impact of B cells in pathogenesis, rituximab (RTX), anti-CD20 monoclonal antibody targeting B cells, has become an effective treatment for AAV and lymphoma by depleting B cells, with high safety and tolerance. B cell depletion occurs through the induction of antibody-dependent cytotoxicity, complement-dependent cytotoxicity and apoptosis [12]. However, the effect of long-term rituximab treatment on maintenance of remission is not clear, and the combination of long-term hormone therapy can bring a series of side effects. Some clinical studies suggest that rituximab combined with CYC may provide a basis for early corticosteroid withdrawal in AAV [13]. In addition, the stimulator of B cell growth, BAFF, has been found to be a promising target for the treatment of AAV. It is thought that regulating the concentration of BAFF after B cell depletion may better increase the efficacy of targeting B cells and reduce the production of autoantibodies. Belimumab is an antagonistic antibody against BAFF. The dual immunotherapy of RTX combined with Belimumab has therapeutic advantages in theory, but the relevant clinical experience and research are still insufficient, and more evidence is needed to support it [14].

Despite the increasing level of diagnosis and treatment, making AAV a chronic recurrent disease, a significant proportion of patients still have an acute onset and poor prognosis [9]. A meta-analysis on the prognosis of AAV proves that the risk of death is at least 2.7 times higher compared to the general population [15]. Therefore, early and correct identification of risk factors is important to assess their condition and improve AAV. Some prove CVD, malignancy, and renal death can be risk factors for premature death from AAV [16]. Other have suggested that renal function, disease activity state and age are important predictors of prognosis in AAV [9]. However, the predictors of adverse outcomes (including death, malignancy, renal replacement therapy (RRT), etc.) occurring in the Chinese AAV population explored by long follow-up have not been fully investigated, and the prognostic factors affected by different inclusion criteria and disease types, so this study is designed to improve the understanding of mortality in these patients and to identify risk factors associated with poorer prognosis.

## Materials and methods

### Patients

Included were 89 patients with initial diagnosis and treatment of AAV from January 2008 to December 2020 in Shanghai Tongji Hospital. All patients met the criteria of the 2012 revised Chapel Hill Consensus Conference (CHCC) [17] or the American College of Rheumatology/European League Against Rheumatism (ACR/EULAR) 2021 criteria [18–20] for the classification as MPA, granulomatosis with polyangiitis (GPA) and eosinophilic granulomatosis with polyangiitis (EGPA). We excluded patients with IgA nephropathy, secondary vasculitis, such as pharmaceutical, infectious and those who combined with other connective tissue diseases, such as Sjogren’s syndrome, systemic lupus erythematosus, and rheumatoid arthritis. Patients were followed up by telephone or outpatient clinic from the initial diagnosis of AAV to the time of their occurrence of death, loss of follow-up, or January 31, 2022, and recorded the type and timing of high-risk outcomes. High-risk outcomes were regarded as the occurrence of death, RRT, and oncologic events, while other AAV patients were included in low-risk group. Information about deaths was taken from medical records in the medical system of Shanghai Tongji Hospital. When data were not available in the medical records, family members were contacted by telephone follow-up.

### Data collection

The following data were collected from AAV patients at first diagnosis and treatment: age, sex, smoking and alcohol consumption, general condition (blood pressure, temperature, weight change), organ involvement (skin, mucosa, chest, ear, nose and throat (ENT), cardiovascular, gastrointestinal, kidney, nervous system) and laboratory data included routine blood and urine, liver and kidney function, electrolyte levels, inflammatory parameters, immunoglobulins, complement, ANCA serology and renal pathology. Cytoplasmic and perinuclear antibodies (c-ANCA and p-ANCA) were measured by indirect immunofluorescence (IIF). Anti-MPO/PR3 antibodies were measured by immunoblotting method. All symptoms and comorbidities diagnoses met the criteria of the Birmingham Vasculitis Activity Score (BVAS) [21]. Routine tests and immunological examinations were done in the testing laboratory of Shanghai Tongji Hospital. Renal function was assessed to estimated glomerular filtration rate (eGFR) using a version of the Chronic Kidney Disease Epidemiology Collaboration (CKD-EPI) equation [22]. The BVAS was calculated by the treating clinician at first diagnosis, when scores ≥ 15 were considered to be in an active state [21]. We also collected the dose of medication which included the first glucocorticoid (GC) pulse therapy and the cumulative dose of CYC at the last follow-up.

Coagulation abnormalities were defined as D-dimer or fibrinogen abnormalities. CKD stagings were according to GFR [23]. RRT was considered as treated with peritoneal dialysis, hemodialysis, renal transplantation, or combined plasma exchange in ESRD.

### Statistical analysis

Person years (PY) were the sum of the follow-up time from the date of AAV diagnosis to death or the end of the study on January 31, 2022, for all patients. All statistical analyses were performed using IBM SPSS Statistics 26.0. Quantitative variables were expressed as mean ± standard deviation (SD) or median and interquartile spacing (IQR), using independent samples *t* test, one-way analysis of variance (ANOVA) or nonparametric tests to compare differences between groups. Categorical variables were compared by chi-square test or Fisher’s exact test. Logistic regression analysis explored the relationship between two variables and calculated the odds ratio (OR) to assess the risk of each variable in groups. Patient survival was analyzed by Kaplan–Meier (K–M) survival analysis, and log-rank tests were used to assess survival differences. Multivariate Cox risk models were performed to investigate the effect of multiple factors on survival time. We used R language to calculate the hypothesis test related to the Cox regression model in the early stage, as detailed in the supplementary material. *P* values less than 0.05 were considered statistically significant with 95% confidence intervals.

## Result

### Patient characteristics

Eighty-nine AAV patients were enrolled. Their characteristics are shown in Table 1. Forty patients (44.9%) with AAV were female, with a mean age of 69.5 years at the time of vasculitis diagnosis and a median follow-up of 3 years (range 0.5 months–11 years). Seventy-four patients (88.1%) had MPA and 69 patients were MPO positive (77.5%), 28 patients underwent renal pathology biopsy with 72.0% of the pathology showing no complement deposits in the kidney. During a 267 PY follow-up period, a total of 46 patients (51.7%) had high-risk events, including 20 patients receiving RRT, no patients undergoing renal transplantation, 12 patients who developed tumors, and 29 patients dying. Twenty-one patients received hormone pulse therapy at their first diagnosis, and 56 patients received cumulative CYC doses of 0.4–16 g. The median cumulative CYC dose for patients with high-risk events was 0.8 g (IQR 0–9).

**Table 1:** Baseline characteristics of 89 AAV patients: differences between patients in the low-risk group and high-risk group

Variable	All patients (*N* = 89)	High-risk group (*N* = 46, 51.7%)	Low-risk group (*N* = 43, 48.3%)	*P* value^1^	OR	*P* value^2^
Age at diagnosis, *x* ± *s*, years	69.5 ± 13.4	72.2 ± 11.1	66.7 ± 15.1	ns		
Female, *n* (%)	40 (44.9)	21(45.7)	19(44.2)	ns		
Diagnosis, *n* (%)				ns		
MPA	74 (88.1)	39 (86.7)	35 (89.7)			
GPA	9 (10.7)	6 (13.3)	3(7.7)			
EGPA	1 (1.2)	0 (0.0)	1 (2.6)			
BVAS, M (P_25_-P_75_)	13 (10–18)	15 (11.8–18)	12 (7.0–16.0)	0.029		
SBP, x ± s, mmHg	135.4 ± 20.1	140.0 ± 23.9	130.6 ± 13.9	0.025		
General, *n* (%)						
Myalgia	8 (9.0)	0 (0.0)	8 (18.6)	0.007		
Arthralgia or arthritis	11 (12.4)	4 (8.7)	7 (16.3)	ns		
Fever	52 (58.4)	23 (50.0)	29 (67.4)	ns		
Emaciation	18 (20.2)	8 (17.4)	10 (23.3)	ns		
Organ involvement, *n* (%)						
Skin	13 (14.6)	3 (6.5)	10 (23.3)	0.025		
Mucous membranes/eyes	10 (11.2)	1 (2.2)	9 (20.9)	0.014		
ENT	23 (25.8)	11 (23.9)	12 (27.9)	ns		
Lung	57 (64.0)	27 (58.7)	30 (69.8)	ns		
Cardiovascular	43 (48.3)	30 (65.2)	13 (30.2)	0.001		
Gastrointestinal	2 (2.2)	1 (2.2)	1 (2.3)	ns		
Renal	72 (80.9)	43 (93.5)	29 (67.4)	0.002		
Hematuria	53 (59.6)	33 (71.7)	20 (46.5)	0.015		
Proteinuria	58 (85.3)	30 (90.9)	28 (82.4)	ns		
Nervous system	10 (11.2)	7 (15.2)	3 (7.0)	ns		
Lab data, *x* ± *s*, M(P_25_-P_75_)/*n* (%)						
Hemoglobin (g/L)	96.7 ± 22.4	89.0 ± 23.0	105.0 ± 18.7	0.001		
RBC (*10^12/L)	3.4 ± 0.8	3.1 ± 0.9	3.6 ± 0.7	0.003		
LYC (*10^9/L)	1.0 (0.6–2.2)	0.9 (0.6–1.5)	1.1 (0.7–1.3)	0.022		
PLT (*10^12/L)	251.7 ± 109.8	228.5 ± 103.4	277.1 ± 112.1	0.037		
Albumin (g/L)	29.5 ± 8.3	27.6 ± 7.4	31.6 ± 8.9	0.023		
Serum sodium (mmol/L)	138.5 ± 4.6	137.8 ± 5.2	139.3 ± 3.8	ns		
Serum calcium (mmol/L)	2.1 ± 0.2	2.0 ± 0.2	2.1 ± 0.1	0.010		
CRP (mg/L)	64.7 (9.1–118.0)	43.2 (4.4–106.7)	71.4(50.9–155.0)	ns		
ESR (mm/h)	64.2 ± 32.8	61.3 ± 31.2	67.0 ± 34.5	ns		
S-creatinine (umol/L)	231.7 ± 232.5	355.0 ± 266.6	99.9 ± 52.2	< 0.001		
BUN (mmol/L)	14.3 ± 11.1	19.8 ± 12.0	8.3 ± 5.9	< 0.001		
eGFR-EPI (mL/(min*1.73 m^2))	29.8 (10.6–69.7)	12.1 (8.2–27.3)	57.6 (34.9–85.1)	< 0.001	0.961	< 0.001
Urine protein positive	40 (46.0)	23 (51.1)	17 (40.5)	ns		
BNP (pg/ml)	204.3 (83.4–497.9)	267.9(118.7–989.1)	100.7 (44.4–247.5)	< 0.001		
Troponin (ng/mL)	0.02 (0.01–0.06)	0.02 (0.01–0.07)	0.01 (0.01–0.03)	0.029		
D-Dimer (mg/L)	2.1 (0.9–4.3)	2.0 (1.0–4.2)	2.4 (0.8–4.6)	0.038		
IgE (IU/mL)	115.0 (41.1–492.0)	137.0 (39.8–534.3)	82.0 (29.0–351.5)	ns		
C3 (g/L)	1.0 ± 0.2	0.9 ± 0.2	1.1 ± 0.3	0.001	0.059	0.019
C4 (g/L)	0.2 ± 0.1	0.2 ± 0.1	0.2 ± 0.1	ns		
p-ANCA positive	69 (78.4)	36 (80.0)	33 (76.7)	ns		
c-ANCA positive	8 (9.0)	5 (11.1)	3 (7.0)	ns		
MPO antibody positive	69 (77.5)	37 (80.4)	32 (74.4)	ns		
PR3 antibody positive	11 (12.5)	6 (13.3)	5 (11.6)	ns		
CYC therapy, *n*(%)	56 (62.9)	31 (67.4)	25(58.1)	ns		

*AAV* ANCA-associated vasculitis; *MPA* Microscopic polyangiitis; *GPA* granulomatosis with polyangiitis; *EGPA* eosinophilcgranulomatosis with polyangiitis; *BVAS* Birmingham vasculitis activity score; *SBP* systolic blood pressure; *ENT* ear, nose and throat; *RBC* red blood cell; *LYC* lymphocyte count; *PLT* platelet; *CRP* C-reactive protein; ESR erythrocyte sedimentation rate; *BUN* blood urea nitrogen; *eGFR* estimated glomerular filtration rate; *BNP* type B natriuretic peptide; *IgE* immunoglobulin E; *C3* complement 3; *C4* complement 4; *p-ANCA* p-antineutrophil cytoplasmic antibodies; *c-ANCA* c-antineutrophil cytoplasmic antibodies; *MPO* myeloperoxidase; *PR3* proteinase 3; *CYC* cyclophosphamide

^1^*p* values obtained with the Chi-square test, the Independent samples *t* test or the Mann–Whitney U test

^2^*p* values obtained with the Multi-factor logistic regression analysis

### Predictors of high-risk outcome

Table 1 shows that there were statistically significant differences in many aspects, such as hypertension, cardiac, renal, ocular/mucosal involvement, renal function between high-risk and low-risk group (*P* < 0.050). By binary regression analysis, eGFR (OR = 0.961, *P* < 0.001) and complement 3 (C3) level (OR = 0.059, *P* = 0.019) were independently associated with high-risk outcomes, higher eGFR and C3 levels reduced the probability of high-risk outcomes.

### Characteristics of high-risk outcome in different subgroups

The study used one-way ANOVA, nonparametric test, chi-square test with multiple comparisons of samples to analyze the characteristics of AAV patients among different high-risk outcomes. It found that age, renal involvement, hemoglobin, red blood cell count, and renal function levels at diagnosis showed significant differences between different prognoses. As seen in the table, the mean value of age was higher than that of the RRT group, with no difference between the two remaining outcomes; in RRT group, hemoglobin, red blood cell count, and eGFR were the lowest, while the mean values of serum creatinine (Scr), blood urea nitrogen (BUN) were higher than those of the other two groups (*P* < 0.050) (Supplementary Table 1).

### Predictors of tumor

After comparing the differences in variables, it was discovered that overall AAV patients had normal or low potassium levels and only blood potassium levels reached significance between tumor group and non-tumor group (*P* = 0.025) (Supplementary Figure 1). After correcting for age, sex, Scr and eGFR, regression analysis still indicated that patients with low blood potassium were more likely to develop tumors in AAV(OR = 0.234, *P* = 0.033) (Supplementary Table 2). We used ROC curves to validate the clinical prediction model, finding serum potassium had a moderate predictive effect on tumor outcome (AUC = 0.7002; *P* = 0.026) (Supplementary Figure 1).

### Predictors of RRT

In Table 2, many variables between RRT group and non-RRT group were different, such as high disease activity, organ involvement, hematologic compromise, and cardiac and renal insufficiency (including type B natriuretic peptide (BNP), BUN, Scr, and eGFR) (*P* < 0.050). Immunoglobulin A (IgA) and C3 levels and other immune indexes in RRT group were significantly lower than those in non-RRT group. Throughout multivariate COX regression analysis after screening variables by the forward: LR method revealed that the probability of renal outcome increased by 29% (HR = 1.290, *P* = 0.006) when the variable BVAS increased by 1 unit, while the probability of renal replacement therapy decreased by 21.8% and 6.6% when the eGFR and total complement level increased by 1 unit, respectively (HR = 0.782, *P* = 0.001; HR = 0.934, *P* = 0.007).

**Table 2:** Characteristics of AAV patients with different renal treatment outcomes

Variable	RRT group (*N* = 20, 22.5%)	Non-RRT group (*N* = 69, 77.5%)	*P* value^1^	HR (95%CI)	*P* value^2^
Age at diagnosis, *x* ± *s*, years	68.4 ± 13.4	69.9 ± 13.5	ns		
Female, *n* (%)	12 (60.0)	28 (40.6)	ns		
BVAS, M (P_25_-P_75_)	17 (14.5–18.8)	12 (8.0–16.0)	0.001	1.290 (1.075–1.549)	0.006
SBP, *x* ± *s*, mmHg	140.0 ± 26.9	134.1 ± 17.7	ns		
DBP, *x* ± *s*, mmHg	81.8 ± 10.4	77.7 ± 11.7	ns		
General, *n* (%)					
Myalgia	0 (0.0)	8 (11.6)	ns		
Arthralgia or arthritis	1 (5.0)	10 (14.5)	ns		
Fever	10 (50.0)	42 (60.9)	ns		
Emaciation	5 (25.0)	13 (18.8)	ns		
Organ involvement, *n* (%)					
Skin	0 (0.0)	13 (18.8)	ns		
Mucous membranes/eyes	1 (5.0)	9 (13.0)	ns		
ENT	4 (20.0)	19 (27.5)	ns		
Lung	13 (65.0)	44 (63.8)	ns		
Cardiovascular	15 (75.0)	28 (40.6)	0.006		
Gastrointestinal	1 (5.0)	1 (1.4)	ns		
Renal	20 (100.0)	52 (75.4)	0.032		
Hematuria	18 (90.0)	35 (50.7)	0.001		
Proteinuria	14 (87.5)	44 (86.3)	ns		
Nervous system	5 (25.0)	5 (7.2)	ns		
Lab data, *x* ± *s*, M (P_25_-P_75_) /*n* (%)					
Hemoglobin (g/L)	79.2 ± 22.1	101.8 ± 19.8	0.000		
RBC (*10^12/L)	2.7 ± 0.9	3.5 ± 0.8	0.000		
LYC (*10^9/L)	0.9 (0.6–1.3)	1.1(0.6–1.5)	0.048		
PLT (*10^12/L)	179.6 ± 80.5	272.9 ± 108.6	0.001		
Albumin (g/L)	26.7 ± 5.4	30.3 ± 8.9	ns		
Serum sodium (mmol/L)	137.3 ± 5.4	138.9 ± 4.4	ns		
Serum calcium (mmol/L)	2.0 ± 0.2	2.1 ± 0.2	ns		
CRP (mg/L)	28.6 (5.8–98.1)	71.4 (19.2–130.8)	ns		
ESR (mm/h)	53.6 ± 26.5	67.0 ± 33.9	ns		
S-creatinine (umol/L)	539.5 ± 262.1	142.5 ± 121.8	< 0.001		
BUN (mmol/L)	25.6 ± 10.3	11.0 ± 9.0	< 0.001		
eGFR (mL/(min*1.73 m^2))	9.8 ± 5.9	62.3 ± 32.4	< 0.001	0.782 (0.680–0.901)	0.001
Urine protein positive	13 (68.4)	27(39.7)	0.026		
BNP (pg/ml)	912.6 (197.8–1494.6)	143.0(48.4–279.3)	< 0.001		
Troponin (ng/mL)	0.02 (0.008–0.3)	0.02(0.01–0.04)	ns		
D-Dimer (mg/L)	1.8 (1.1–4.2)	2.1(0.8–4.6)	ns		
IgA (g/L)	2.4 ± 1.3	3.2 ± 1.2	0.020		
IgE (IU/mL)	140.9 (35.7–629.3)	115.0 (41.7–351.5)	ns		
TC (g/L)	32.6 ± 14.5	40.0 ± 12.1	0.041	0.934 (0.889–0.982)	0.007
C3 (g/L)	0.9 ± 0.2	1.0 ± 0.3	0.040		
C4 (g/L)	0.2 ± 0.1	0.2 ± 0.1	ns		
p-ANCA positive	15 (78.9)	54 (78.3)	ns		
c-ANCA positive	3 (15.8)	5 (7.2)	ns		
MPO positive	16 (80.0)	53 (76.8)	ns		
PR3 positive	2 (10.5)	9 (13.0)	ns		
CYC therapy, *n*(%)	16 (80.0)	40 (58.0)	ns		
Renal pathology with complement deposition, *n*(%)	3 (42.9)	4 (57.1)	ns		
Tumor, *n*(%)	2 (10.0)	10 (14.5)	ns		
Death, *n*(%)	8 (40.0)	21 (30.4)	ns		

*RRT* renal replacement therapy; *BVAS* Birmingham vasculitis activity score; *SBP* systolic blood pressure; *ENT* ear, nose and throat; *RBC* red blood cell; *LYC* lymphocyte count; *PLT* platelet; *CRP* C-reactive protein; *ESR* erythrocyte sedimentation rate; *BUN* blood urea nitrogen; *eGFR* estimated glomerular filtration rate; *BNP* type B natriuretic peptide; *IgA* immunoglobulin A; *IgE* immunoglobulin E; *TC* total complement; *C3* complement 3; *C4* complement 4; *p-ANCA* p-antineutrophil cytoplasmic antibodies; *c-ANCA* c-antineutrophil cytoplasmic antibodies; *MPO* myeloperoxidase; *PR3* proteinase 3; *CYC* cyclophosphamide

^1^*p* values obtained with the Chi-square test, the Independent samples *t* test or the Mann–Whitney U test

^2^*p* values obtained with the multivariate Cox risk model analysis

### Predictors and causes of mortality

During the follow-up, infection and organ failure due to active vasculitis remained the main cause (Supplementary Figure 2). Compared with the survival group, those with the death outcome were older at diagnosis, more probable to have cardiac, and renal involvement, lower blood calcium levels, more severely impaired renal function (including Scr, BUN, and eGFR), and higher D-Dimer and fibrinogen levels (*P* < 0.050) (Supplementary Table 3). Based on the above-screened variables, we further analyzed the risk factors correlated with mortality. In univariate analysis, older age, cardio involvement at diagnosis, higher fibrinogen, lower blood calcium, and poorer renal function enlarged the probability of death (Table 3). Through multivariate analysis, only age at diagnosis (HR = 1.060, *P* = 0.006), eGFR (HR = 0.982, *P* = 0.018) individually predicted the death.

**Table 3:** Univariate and multivariate Cox risk model analysis of all-cause mortality variables in AAV patients

Variable	Univariable analysis		Multivariable analysis	
	HR (95%CI)	*P* value	HR (95%CI)	*P* value
Age (year)	1.069 (1.024–1.117)^*^	0.003	1.060(1.016–1.105)^*^	0.006
Mucous membranes/eyes	0.038 (0.000–4.233)	0.174		
Cardiovascular	2.248 (1.015–4.976)^*^	0.046		
Renal	31.904 (0.679–1500.142)	0.078		
Proteinuria	29.165 (0.162–5258.155)	0.203		
Hemoglobin (g/L)	0.985 (0.969–1.001)	0.065		
LYC (*10^9/L)	0.539 (0.288–1.009)	0.053		
Serum calcemia (mmol/L)	0.041 (0.005–0.363)^*^	0.004		
BNP (pg/ml)	1.000 (0.999–1.001)	0.965		
IgE (IU/mL)	1.000 (1.000–1.001)	0.136		
PCT (ng/mL)	0.991 (0.883–1.112)	0.879		
D-Dimer (mg/L)	1.026 (0.968–1.087)	0.394		
Fibrinogen (g/L)	1.484 (1.053–2.091)^*^	0.024		
S-creatinine (umol/L)	1.001 (1.000–1.003)^*^	0.010		
BUN (mmol/L)	1.059 (1.029–1.090)^*^	< 0.001		
eGFR (mL/(min*1.73 m^2))	0.978 (0.966–0.991)^*^	0.001	0.982 (0.968–0.997)^*^	0.018

*AAV* ANCA-associated vasculitis; *LYC* lymphocyte count; *BNP* type B natriuretic peptide; *IgE* immunoglobulin E; *PCT* procalcitonin; *BUN* blood urea nitrogen; *eGFR* estimated glomerular filtration rate; *HR* hazard ratio

### Patients and renal survival curves

**Fig. 1:**
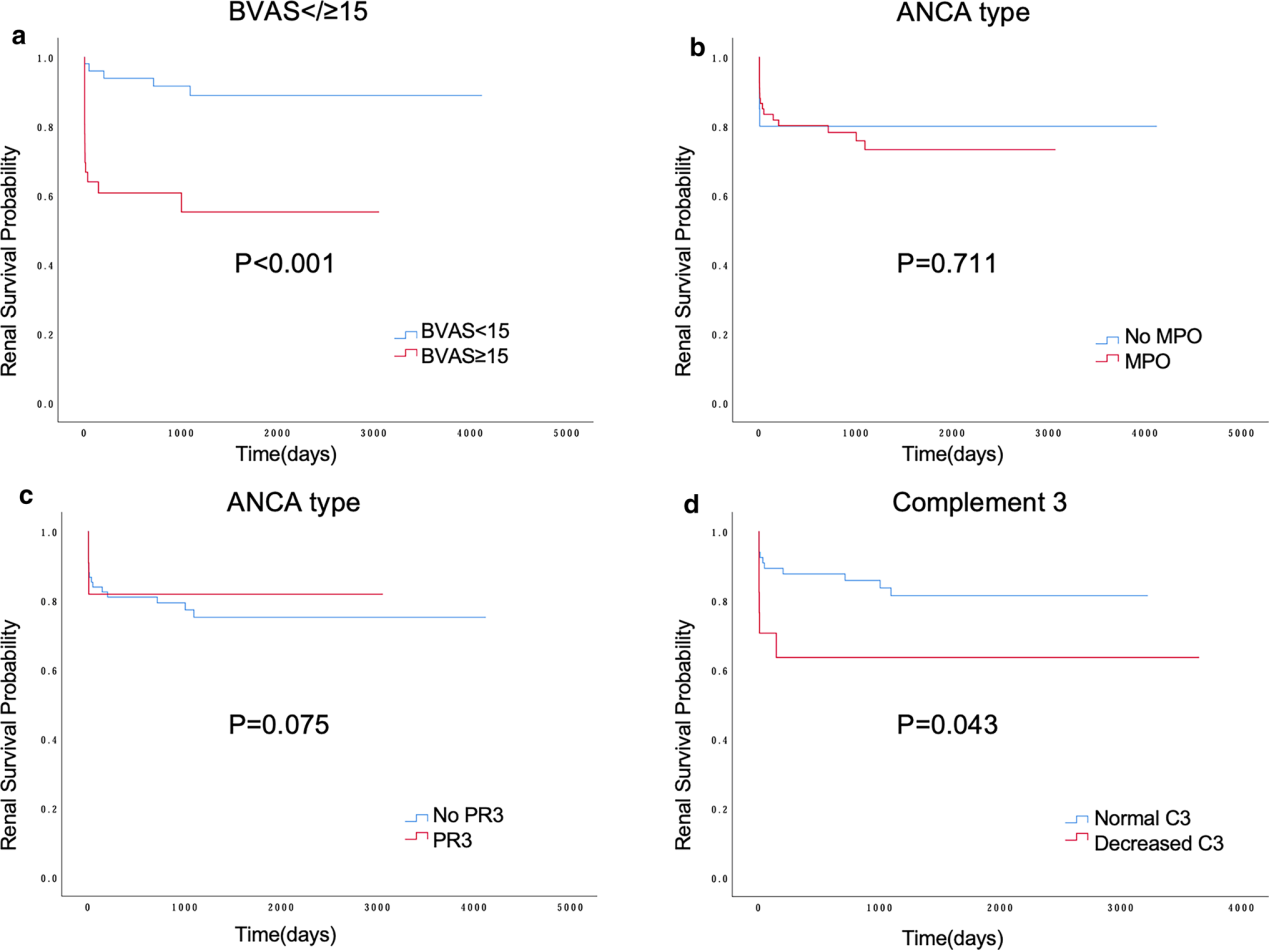
Comparison of renal survival rates between different subgroups in AAV. Renal survival by BVAS score (**a**), MPO (**b**), PR3 (**c**), Complement 3 level (**d**)at diagnosis; p values obtained with the log-rank analysis. *AAV* ANCA-associated vasculitis; *BVAS* Birmingham Vasculitis Activity Score; *MPO* myeloperoxidase; *PR3* proteinase 3

**Fig. 2:**
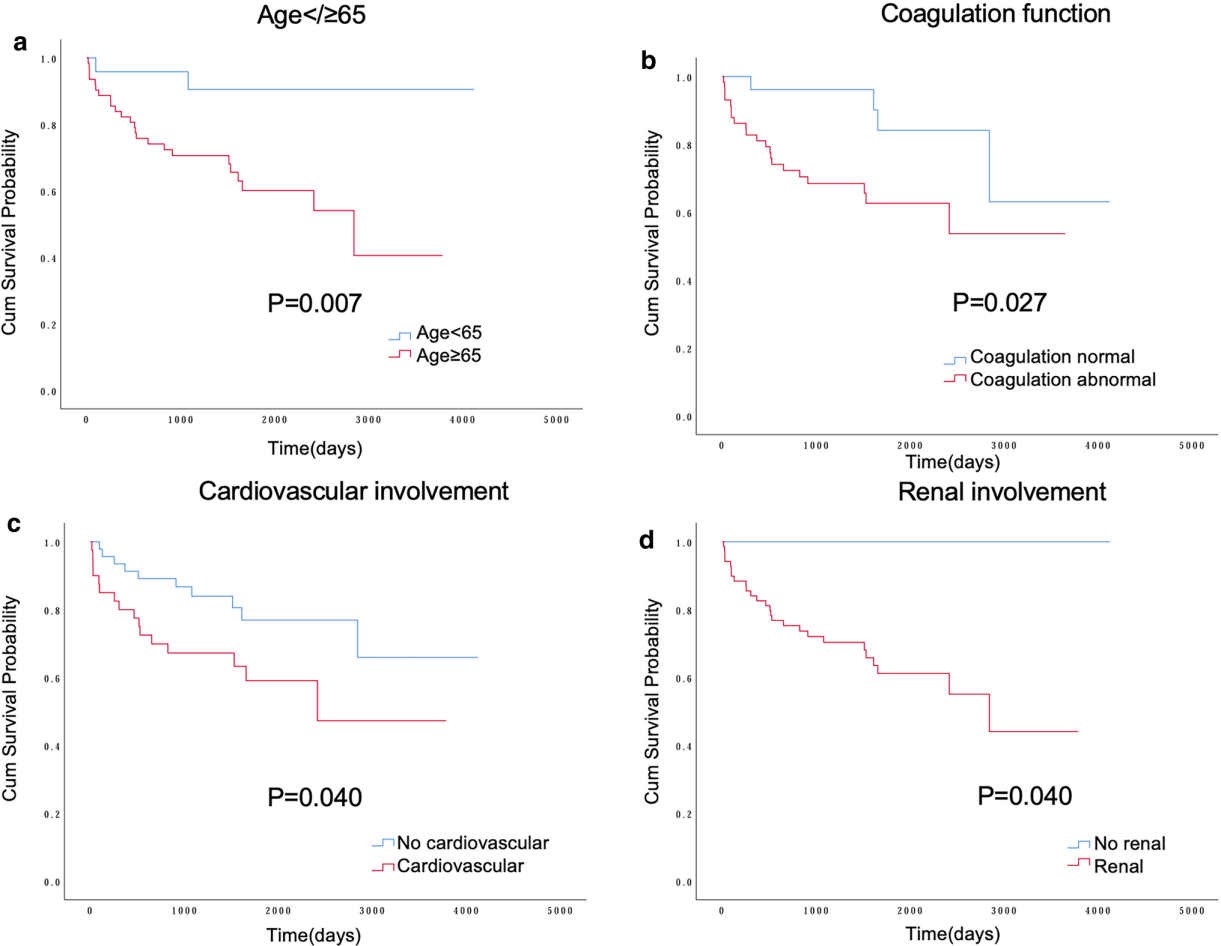
Comparison of cum survival rates between different subgroups in AAV. Cum survival of AAV patients by age at diagnosis (**a**), coagulation (**b**), cardiovascular involvement (**c**), renal involvement (**d**) status; *p* values obtained with the log-rank analysis. *AAV* ANCA-associated vasculitis; *CKD* chronic kidney disease; *RRT* renal replacement therapy

Figure 1 displays renal survival rate between different subgroups in AAV. The higher the BVAS, the poorer the kidney survival (*P* < 0.001). There was no significant difference in renal survival among patients with different ANCA types (*P* > 0.050). AAV patients with cardiac and renal involvement at diagnosis were more likely to develop ESRD later (*P* = 0.002, *P* = 0.014) (Supplementary Figure 3). In Fig. 2, patients aged ≥ 65 years had a higher mortality rate than younger patients (*P* = 0.007). In addition, patients with early coagulation abnormalities, cardiac and renal involvement also had lower cumulative survival than normal patients (*P* < 0.050). However, no difference in survival was found between patients who had renal replacement therapy or not (Supplementary Figure 4).

### Correlations between different treatment and high-risk outcomes

We examined the association between treatment and outcomes in AAV. The cumulative exposure dose for all AAV patients using CYC was less than 36 g. No significant differences were seen in cumulative CYC use between the different high-risk outcomes (*P* > 0.050) (Supplementary Figure 5). After bivariate correlation analysis, there was a medium correlation between high-dose hormone shock therapy at the first visit and RRT (*φ* = 0.525, *P* < 0.001), however, there was little correlation with other outcomes (Supplementary Table 4).

## Discussion

MPA is the main part in China, in which the kidney is the most common organ involved, different from some Caucasian studies [1]. At present, although the immunosuppressive therapy has greatly improved the mortality rate, there are still a part of AAV patients developing tumor, CKD and other adverse events, especially less research has been done on tumors in Chinese patients. Therefore, it is necessary to determine the risk factors for different outcomes. To our knowledge, this is the first long-term study to comprehensively assess the risk factors for Chinese AAV outcomes, including tumor, RRT, and death.

The majority of the cohort consists of MPA patients with MPO positivity, more than half of the patients have a high-risk outcome. No correlation is discovered between prognosis and ANCA type. This is similar to what previous studies have found, renal outcomes were not determined by ANCA specificity [24]. However, the impact of ANCA staging on outcomes in AAV patients remains controversial. In some studies, increased mortality was found in patients with MPO-ANCA. MPO-ANCA has significantly worse renal survival compared to PR3-positive patients, possibly related to the longer disease states and different renal pathological manifestations [6]. But we might find decreased eGFR and C3 levels are independent risk factors for the high-risk outcomes in this study. Many studies supported this idea. AAV patients with low sC3 tend to have lower eGFR and a worse prognosis than patients with normal sC3 [25]. What’s more, decreased C3 levels at diagnosis are also significantly associated with reduced renal survival in this study and 40.0% of AAV patients who underwent renal biopsy show immunoglobulin IgA, IgG, IgM, or C3 deposition in this study. Reduced C3 levels and complement deposition in glomeruli as features of glomerulonephritis were well established before[26]; all these may support a role for neutrophil-activated complement replacement pathways in the pathogenesis of AAV [27, 28]. In particular, complement components, such as C5a and C3a, and neutrophil extracellular traps (NETs) activate the complement system to further amplify the inflammatory pathway, leading to endothelial cell injury and participating in the pathogenesis [29].

We then refine the risk factors for each high-risk event. 13.5% of the AAV patients in the cohort had tumors, including gastrointestinal tract tumors, bladder cancer and so on. Lower serum potassium level is discovered to be a risk factor for developing tumors. Studies proved that an environment of high K + concentration outside tumor cells inhibited the proliferation and activation of T cells, thus increasing the risk of tumor progression and metastasis [30]. Cahalan et al. found potassium channel blockers, especially the voltage-gated potassium channel Kv1.3, which mainly transports K + in T cells could inhibit the proliferation of T lymphocytes [31]. The Kv1.3 channel is also involved in inducing proliferation and apoptosis of expressed cancer cells [32]. Indeed, the Kv1.3 expression disorder was detected in various malignancies [33]; inhibition of Kv1.3 channels located in mitochondria of cancer cells was proved to selectively induce apoptosis in vivo [34]. In addition, selective blocking of Kv1.3 inhibits effector memory CD4 + T cell activity in inflamed tissue, which plays a key role in mitigating AAV progression [35]. Therefore, we hypothesize that the effect of potassium channel on T cells might be involved in the co-pathogenic pathway of AAV and tumor. However, the result of the study is somewhat different from the previous ones; studies reported for the first time that increased K^+^ levels increased the absolute risk of all cancers in general population [36]. It may be due to insufficient sample size or different disease inclusion. Although this study fails to establish a causal relationship between serum potassium and tumor, the current information about the risk factors for the development of tumors in AAV patients is few. Another reason is that the cumulative exposure of CYC to the patients from the onset of the tumor in this study was low and no correlation was found between the dose and tumor, which may allow us to ignore the influence of the drug on tumor development and pay more attention to the interaction between the disease itself and the tumor. These likewise suggest that long-term monitoring of blood potassium levels should be performed in AAV patients, which may be beneficial to early screen tumor.

In the RRT group, we also find that patients have a higher BVAS at diagnosis and more prominent clinical symptoms, mainly in cardiac and renal involvement (presence of hematuria and impaired renal function), which could show a clear relationship between vasculitis activity and RRT. What’s more, this study identified higher BVAS and lower eGFR as major risk factors for renal survival, as previously demonstrated [37]. Microscopic hematuria is an early sign of AAV disease activity, and the disappearance of hematuria is a symbol of disease remission [38]. This is also reflected by the present study, in which the kidney survival rate is significantly lower in patients with disease activity at diagnosis and in those presenting with hematuria than in the normal group. Therefore, early identification of renal involvement is essential to improve renal survival. Interestingly, for RRT, patients in this study do not account for death in the same proportion as non-dialysis patients and have no significance, suggesting that dialysis treatment may be beneficial in stabilizing renal function and reducing vasculitis recurrence [39].

In addition, serum IgA (sIgA) levels draw our attention. In our study, sIgA levels not only are found to be lower for AAV patients undergoing RRT, with a greater proportion of patients in the low-risk group experiencing ocular/mucosal, ENT and chest, while a lower probability of these mucosal occurrences in the death group, suggesting that sIgA may play an active role in alleviating AAV progression. We learn IgA is found to be a local mucosal immune-associated antibody and may be a major driver of autoimmune complex-mediated formation of NETs [40]. IgA-ANCA in AAV can exert anti-inflammatory or pro-inflammatory effects by binding to different Fc receptors (FcRs) [41]. And IgA PR3-ANCA was observed in approximately one quarter of patients with GPA in previous studies and was less common in severe renal disease [42]. Nephropathy with IgA deposition may show lower disease activity than patients with oligoimmune AAV [43]. In MPO-ANCA-associated glomerulonephritis, serum globulin levels were negatively associated with ESRD, but patients with and without IgA deposition did not show differences in renal outcomes [44]. Therefore, the impact of IgA on AAV disease progression is worth thinking about. There are few clinical studies on the correlation between ANCA-associated glomerulonephritis and serum IgA levels, and no more evidence has been found to support the protective role of IgA. As more kidney biopsies show evidence of immune complex deposition, it is worthwhile to explore more the clinical manifestations and immunological characteristics of ANCA-associated nephropathy with immune complex deposition and ANCA with different immunoglobulin species in the future.

Besides tumors and RRT, we also discover a number of deaths. The cumulative survival rates at 1, 3, and 5 years are 86.0%, 76.2%, and 68.6%, similar to other studies on Chinese populations [45]. The important thing about this study is that only age at diagnosis and eGFR level independently predict death in AAV patients. Other studies also highlighted the importance of age and renal function in survival of AAV [9]. Apart from this, COX analysis reflects that a patient at diagnosis with cardiovascular involvement and abnormal coagulation function still has an increased risk of death. Patients with cardiovascular and renal involvement at diagnosis have poorer patients and renal survival rate. The leading causes of death include vasculitis activity and infection, as well as cardiovascular events. Therefore, early screening of cardio and renal function in AAV patients should be performed to improve prognosis. Most current studies prove early cardiovascular involvement may predict the onset of death [16]. We speculate that this may be due to the fact as vasculitis is a chronic inflammatory state that can lead to diffuse endothelial dysfunction, a predictor of atherosclerotic disease, and one of the potential mechanisms is kidney disease [46]. The inflammatory state of AAV not only causes endothelial cell damage, but alters the balance of coagulation and anticoagulation through cytokines [47], which aggravates itself inflammatory state. As renal function deteriorates, the body could contribute to cardiovascular events by affecting metabolic, inflammatory responses, oxidative stress and hemodynamic pathways [48, 49]. On the other hand, kidney injury itself can affect coagulation [50]. In brief, AAV, renal disease, and cardiovascular event interact with each other to likely promote the progression of their respective diseases.

In terms of treatment, all patients in this cohort are exposed to low doses of CYC, either because some patients do not follow the medically prescribed admission, or most patients with renal involvement, who are regularly treated with dialysis in the ESRD, do not continue immunosuppressive therapy. In addition, no significant correlation was found regarding tumor outcome and CYC accumulation, and no serious adverse effects are documented with regard to CYC, as it is possible, due to the low exposure dose. Treatment with cumulative CYC doses < 10 g was not associated with an increased risk of malignancy in previous studies, and only at higher doses was the risk of cancer significantly increased [51]. In this study, there is no specific intervention on treatment, so the effect of CYC on AAV outcomes remains to be considered. A medium correlation is found between the use of high-dose hormone pulse therapy at the first visit and RRT outcomes, that does not mean causality. As we are concerned, the short-term high-dose hormone pulse therapy is mainly used for diseases that cause acute organ damage [52]. Therefore, the use of hormone pulse therapy in patients with first AAV diagnosis is considered to have severe organ involvement and poor long-term prognosis. Only a very small number of patients (less than 5%) in this cohort are treated with RTX. It is true that RTX is rarely used in China, which may be one of the reasons for the high mortality rate in the early stage of this study. On the other hand, most patients are too sick to use biological agents. Unfortunately, it was not possible to study whether RTX would reduce the probability of AAV adverse outcome events in this study. At present, studies at home and abroad have confirmed that rituximab is a good inducer of remission [53]. The guidelines recommend that glucocorticoids, CYC or RTX should be combined to induce remission therapy in severe AAV [54]. Many new B-cell therapies for AAV are underway, but further studies and evidence are needed for efficacy and long-term toxicity in clinical studies of AAV patients. For example, second-generation anti-CD20 antibodies, such as Obinutuzumab, have increased antibody-dependent cytotoxicity and greater direct B-cell killing capacity than rituximab; dual immunotherapy has become a research hotspot, which can achieve a wider range of B cell inhibition and enhance B cell depletion than RTX alone [55].

There are some superiorities and limitations to our research. It is a long-term follow-up prospective study of AAV population in China. Many clinical indicators were included in the early stage, and the association between different poor prognosis and clinical characteristics of AAV was relatively completely studied. But this is a study from a single center. The sample size is a big concern, due to the low incidence of AAV who need to be hospitalized. In addition, AAV classification was not fully studied, considering that most AAV types in this study are MPA, which is coordinated with other studies in China, a detailed grouping to compare the differences in ANCA classifications may lead to insufficient sample size and increased statistical error. Those limitations could be improved by a multiple-center study in our further work.

## Conclusion

In conclusion, most MPA patients with MPO positive are associated with renal damage at diagnosis, and the long-term prognosis remains poor. Decreased eGFR and C3 levels may be predictors of poor prognosis in AAV. The combination of BVAS and eGFR is a great prognosticator for RRT outcome. Age at diagnosis and eGFR can independently predict the death. Whether potassium level exacerbates the risk of cancer development in AAV and the extent to which serum immunoglobulin concentrations affect renal outcomes need to be further explored in the future.

## Supplementary Information

Below is the link to the electronic supplementary material.

Supplementary file1 (PDF 1945 KB)
