# Supplementary material for: Predictors of poor prognosis in ANCA-associated vasculitis (AAV): a single-center prospective study of inpatients in China
Source: Clin Exp Med. 2022 Oct 16;23(4):1331–43. doi: 10.1007/s10238-022-00915-z (PMC10390347; doi:10.1007/s10238-022-00915-z)
Supplement: Supplementary file 1 — Supplementary file1 (PDF 1945 KB) [file 10238_2022_915_MOESM1_ESM.pdf]

Predictors of Poor Prognosis in ANCA-Associated Vasculitis (AAV): A single-center prospective study of inpatients in China

Authors: Ronglin Gao[#1], Zhenzhen Wu[#1], Xianghuai Xu[#2], Jincheng Pu[1], Shengnan Pan[1], Youwei Zhang[1], Shuqi Zhuang[1], Lufei Yang[1], Yuanyuan Liang[1], Jiamin Song[1], Jianping Tang*[1], Xuan Wang*[1]

[#]Those authors have contributed equally to this work.

Affiliation

1 Department of Rheumatology and Immunology, Tongji Hospital, School of Medicine, Tongji University, No. 389 Xincun Road, Shanghai 200065, China.

2 Department of Pulmonary and Critical Care Medicine, Tongji Hospital, School of Medicine, Tongji University, No. 389 Xincun Road, Shanghai 200065, China.

* **Correspondence:** Jianping Tang and Xuan Wang.

Xuan Wang: Department of Rheumatology and Immunology, Tongji Hospital, School of Medicine, Tongji University, No. 389 Xincun Road, Shanghai 200065, China. E-mail: xuan2018@tongji.edu.cn.

Jianping Tang: Department of Rheumatology and Immunology, Tongji Hospital, School of Medicine, Tongji University, No. 389 Xincun Road, Shanghai 200065, China. E-mail: tangjp6512@126.com.

Supplementary material 1

Table 1 Variables comparison between subgroups of high-risk events in AAV patients

Variable	Group0 Tumor	Group1 RRT	Group2 Death	P-value
	N=12	N=20	N=29	
**Age at diagnosis, x±s, years**	70.3±9.2	68.4±13.4	76.1±7.9[*]	0.030[b]
**Female, n (%)**	5(41.7)	12(60.0)	10(34.5)	ns
**BVAS≥15 at diagnosis, n (%)**	5(41.7)	15(75.0)	13(44.8)	ns
**General, n (%)**				
Myalgia	0(0.0)	0(0.0)	0(0.0)	ns
Arthralgia or arthritis	1(8.3)	1(5.0)	3(10.3)	ns
Fever	5(41.7)	10(50.0)	15(51.7)	ns
Emaciation	2(16.7)	5(25.0)	3(10.3)	ns
**Organ involvement, n (%)**				
**Skin**	1(8.3)	0(0.0)	2(6.9)	ns
**Mucous membranes/eyes**	0(0.0)	1(5.0)	0(0.0)	ns
**ENT**	4(33.3)	4(20.0)	7(24.1)	ns
**Lung**	9(75.0)	13(65.0)	16(55.2)	ns
**Cardiovascular**	6(50.0)	15(75.0)	19(65.5)	ns
**Gastrointestinal**	0(0.0)	1(5.0)	0(0.0)	ns
**Renal**	9(75.0)[#]	20(100.0)	29(100.0)	0.007[a]
Hematuria	8(66.7)	18(90.0)	21(72.4)	ns
Proteinuria	8(88.9)	14(87.5)	20(100.0)	ns
**Nervous system**	3(25.0)	5(25.0)	4(13.8)	ns
**Lab data, x±s /n (%)**				ns
Hemoglobin (g/L)	107.7±20.5[*#]	79.2±22.1	89.6±18.2	0.001[b]
RBC (*10^12/L)	3.6±0.8	2.7±0.9[§]	3.1±0.8	0.010[b]
PLT (*10^12/L)	259.8±79.0	179.6±80.5	247.2±110.5	0.028[b]
Serum kalcium (mmol/L)	3.4±0.5	3.9±0.7	3.8±0.8	ns
CRP (mg/L)	67.7±80.3	76.8±78.5	88.4±62.0	ns
ESR (mm/h)	59.2±36.7	53.6±26.5	72.3±32.1	ns
S-creatinine (umol/L)	211.3±206.5	539.5±262.1[§#]	383.1±267.9	0.001[b]
BUN (mmol/L)	12.9±12.1	25.6±10.3[§]	21.8±12.6	0.017[b]
eGFR (mL/(min*1.73m^2))	54.4±36.6	9.8±5.9[§#]	27.8±26.8	0.001[b]
Urine protein positive	6(50.0)	13(68.4)	16(55.2)	ns
BNP (pg/ml)	428.6±406.6	978.3±1083.8	418.0±501.1	ns
Troponin (ng/mL)	0.02±0.02	0.4±0.9	0.1±0.4	ns
D-Dimer (mg/L)	4.6±9.8	5.3±6.1	4.7±4.2	ns
Fibrinogen (g/L)	3.6±1.0	4.1±1.1	4.3±1.0	ns
IgA (g/L)	2.9±1.1	2.4±1.3	3.2±1.2	ns
C3 (g/L)	0.9±0.2	0.9±0.2	0.9±0.2	ns

C4 (g/L)	0.2±0.1	0.2±0.1	0.2±0.1	ns
p-ANCA positive	11(91.7)	15(78.9)	24(85.7)	ns
c-ANCA positive	1(8.3)	3(15.8)	2(7.1)	ns
MPO positive	10(83.3)	16(80.0)	24(82.8)	ns
PR3 positive	3(25.0)	2(10.5)	3(10.7)	ns

The other p-values (§p<0.05 vs. group 0; *p<0.05 vs. group 1; #p<0.05 vs. group 2) indicate pairwise multiple-comparisons performed with the test; a *p*-values obtained with the Chi square test; b *p*-values obtained with the Kruskall-Wallis H test or one-way ANOVA.

Abbreviations: RRT: renal replacement therapy; BVAS: Birmingham Vasculitis Activity Score; ENT: ear, nose and throat; RBC: red blood cell; PLT: platelet; CRP: C-reactive protein; ESR: erythrocyte sedimentation rate; BUN: blood urea nitrogen; eGFR: estimated glomerular filtration rate; BNP: type B natriuretic peptide; IgA: immunoglobulin A; C3: complement 3; C4: complement 4; p-ANCA: p-antineutrophil cytoplasmic antibodies; c-ANCA: c-antineutrophil cytoplasmic antibodies; MPO: myeloperoxidase; PR3: proteinase 3.

Supplementary material 2

Table 2 Analysis of associated factors between the tumor and non-tumor groups

Variable	Tumor group (N=12, 13.5%)	No-tumor group (N=77, 86.5%)	P-value[1]	OR	P-value[2]
**Age at diagnosis, x±s, years**	70.3±9.2	69.3±14.0	ns		
**Female, n (%)**	5(41.7)	35(45.5)	ns		
**BVAS≥15 at diagnosis, n (%)**	5(41.7)	31(40.3)	ns		
**General, n (%)**					
Myalgia	0(0.0)	8(10.4)	ns		
Arthralgia or arthritis	1(8.3)	10(13.0)	ns		
Fever	5(41.7)	47(61.0)	ns		
Emaciation	2(16.7)	16(20.8)	ns		
**Organ involvement, n (%)**					
**Skin**	1(8.3)	12(15.6)	ns		
**Mucous membranes/eyes**	0(0.0)	10(13.0)	ns		
**ENT**	4(33.3)	19(24.7)	ns		
**Lung**	9(75.0)	48(62.3)	ns		
**Cardiovascular**	6(50.0)	37(48.1)	ns		
**Gastrointestinal**	0(0.0)	2(2.6)	ns		
**Renal**	9(75.0)	63(81.3)	ns		
Hematuria	8(66.7)	45(58.4)	ns		
Proteinuria	8(88.9)	50(86.2)	ns		
**Nervous system**	3(25.0)	7(9.1)	ns		
**Lab data, x±s, M(P_{25}-P_{75}) /n (%)**					
Hemoglobin (g/L)	107.7±20.5	95.0±22.3	ns		
LYC (*10^9/L)	1.5(0.7-1.8)	0.9(0.6-1.3)	ns		
PLT (*10^12/L)	259.8±79.0	250.4±114.2	ns		
Serum sodium (mmol/L)	137.1±4.1	138.8±4.7	ns		
Serum calcium (mmol/L)	2.0±0.2	2.1±0.2	ns		
Serum potassium (mmol/L)	3.4±0.5	3.8±0.6	0.025	0.234	0.033
CRP (mg/L)	6.9(2.6-123.3)	73.4(16.8-118.0)	ns		
ESR (mm/h)	59.2±36.7	65.0±32.3	ns		
S-creatinine (umol/L)	211.3±206.5	234.9±237.4	ns		
BUN (mmol/L)	12.9±12.1	14.5±11.0	ns		
eGFR-EPI (mL/(min*1.73m^2))	38.2(10.6-84.2)	28.4(10.6-62.4)	ns		
BNP (pg/ml)	89.8(24.7-674.6)	210.1(97.9-529.8)	ns		
Troponin (ng/mL)	0.02(0.01-0.05)	0.02(0.01-0.07)	ns		
D-Dimer (mg/L)	1.8(0.3-2.2)	2.5(0.9-5.2)	ns		
IgE (IU/mL)	52.5(28.0-137.0)	161.5(45.8-534.3)	ns		
C3 (g/L)	0.9±0.2	1.0±0.3	ns		
C4 (g/L)	0.2±0.1	0.2±0.1	ns		

p-ANCA positive	11(91.7)	58(76.3)	ns
c-ANCA positive	1(8.3)	7(9.2)	ns
MPO positive	10(83.3)	59(76.6)	ns
PR3 positive	3(25.0)	8(10.5)	ns
**CYC therapy, n(%)**	8(66.7)	48(62.3)	ns
**RRT, n (%)**	2(16.7)	18(23.4)	ns
**Death, n (%)**	5(41.7)	24(31.2)	ns

1p-values obtained with the Chi square test, the Independent samples t-test or the Mann-Whitney U test; 2p-values obtained with the Multi-factor logistic regression analysis.

Abbreviations: BVAS: Birmingham vasculitis activity score; ENT: ear, nose and throat; LYC: lymphocyte count; PLT: platelet; CRP: C-reactive protein; ESR: erythrocyte sedimentation rate; BUN: blood urea nitrogen; eGFR: estimated glomerular filtration rate; BNP: type B natriuretic peptide; IgE: immunoglobulin E; C3: complement 3; C4: complement 4; p-ANCA: p-antineutrophil cytoplasmic antibodies; c-ANCA: c-antineutrophil cytoplasmic antibodies; MPO: myeloperoxidase; PR3: proteinase 3; CYC: cyclophosphamide; GC: glucocorticoid; RRT: renal replacement therapy.

Supplementary material 3

Table 3 Baseline characteristics between the death and survival groups

Variable	Death group (N=29, 32.6%)	Survival group (N=60, 67.4%)	P-value
**Age at diagnosis, x±s, years**	76.1±7.9	66.3±14.3	0.000
**Female, n (%)**	10(34.5)	30(50.0)	ns
**BVAS ≥15 at diagnosis, n (%)**	13(44.8)	23(38.3)	ns
**General, n (%)**			
Myalgia	0(0.0)	8(13.3)	ns
Arthralgia or arthritis	3(10.3)	8(13.3)	ns
Fever	15(51.7)	37(61.7)	ns
Emaciation	3(10.3)	15(25.0)	ns
**Organ involvement, n (%)**			
**Skin**	2(6.9)	11(18.3)	ns
**Mucous membranes/eyes**	0(0.0)	10(16.7)	0.048
**ENT**	7(24.1)	16(26.7)	ns
**Lung**	16(55.2)	41(68.3)	ns
**Cardiovascular**	19(65.5)	24(40.0)	0.024
**Gastrointestinal**	0(0.0)	2(3.3)	ns
**Renal**	29(100.0)	43(71.7)	0.001
Hematuria	21(72.4)	32(53.3)	ns
Proteinuria	20(100.0)	38(80.9)	ns
**Nervous system**	4(13.8)	6(10.0)	ns
**Bronchiectasis**	5(17.2)	9(15.0)	ns
**Lab data, x±s, M(P_{25}-P_{75}) /n (%)**			
Hemoglobin (g/L)	89.6±18.2	100.2±23.5	0.036
LYC (*10^9/L)	0.8(0.5-1.4)	1.1(0.7-1.6)	0.027
PLT (*10^12/L)	247.2±110.5	253.9±110.3	ns
Serum sodium (mmol/L)	137.6±5.3	139.0±4.3	ns
Serum calcium (mmol/L)	2.0±0.2	2.1±0.2	0.006
CRP (mg/L)	89.7(26.6-120.0)	60.0(4.9-107.8)	ns
ESR (mm/h)	72.3±32.1	60.7±32.7	ns
PCT (ng/mL)	0.2(0.1-1.1)	0.1(0.1-0.4)	0.042
S-creatinine (umol/L)	346.3±242.6	176.4±207.7	0.001
BUN (mmol/L)	21.8±12.6	10.6±8.2	<0.001
eGFR (mL/(min*1.73m^2))	10.7(8.2-22.0)	42.8(19.1-77.5)	<0.001
BNP (pg/ml)	267.9(147.6-940.6)	119.2(44.4-468.3)	0.043
Troponin (ng/mL)	0.03(0.01-0.07)	0.01(0.01-0.03)	ns
D-Dimer (mg/L)	2.5(1.9-7.4)	1.7(0.8-3.6)	0.010
Fibrinogen (g/L)	4.3±1.0	3.7±1.2	0.033
IgE (IU/mL)	169.5(75.4-674.5)	80.6(19.7-351.5)	0.013

C3 (g/L)	0.9±0.2	1.0±0.3	ns
C4 (g/L)	0.2±0.1	0.2±0.1	ns
p-ANCA positive	24(85.7)	45(73.8)	ns
c-ANCA positive	2(7.1)	6(10.0)	ns
MPO positive	24(85.7)	45(75.0)	ns
PR3 positive	3(10.7)	8(13.3)	ns
**CYC therapy, n(%)**	19(65.5)	37(61.7)	ns
**Tumor, n (%)**	5(17.2)	7(11.7)	ns
**RRT, n (%)**	8(27.6)	12(20.0)	ns

Abbreviations: BVAS: Birmingham vasculitis activity score; ENT: ear, nose and throat; LYC: lymphocyte count; PLT: platelet; CRP: C-reactive protein; ESR: erythrocyte sedimentation rate; PCT: procalcitonin; BUN: blood urea nitrogen; eGFR: estimated glomerular filtration rate; BNP: type B natriuretic peptide; IgE: immunoglobulin E; C3: complement 3; C4: complement 4; p-ANCA: p-antineutrophil cytoplasmic antibodies; c-ANCA: c-antineutrophil cytoplasmic antibodies; MPO: myeloperoxidase; PR3: proteinase 3; CYC: cyclophosphamide; GC: glucocorticoid; RRT: renal replacement therapy.

Supplementary material 4

Table 4 Relationship between high-dose GC pulse therapy and different outcomes

Outcome	GC shock therapy<br>N=21, 23.6%	No-GC shock therapy<br>N=68, 76.4%	P-value[1]	***φ***	P-value[2]	95%CI
High-risk, n(%)	16(76.2)	30(44.1)	0.010	0.273	0.010	0.009-0.013
RRT, n(%)	13(61.9)	7(10.3)	<0.001	0.525	<0.001	0.000-0.00003
Tumor, n(%)	2(9.5)	10(14.7)	0.809			
Death, n(%)	8(38.1)	21(30.9)	0.538			

1p-values obtained with the Chi square test; 2 p-values obtained with the Relevance test.

Abbreviations: ***φ***: Phi coefficient; GC: glucocorticoid; RRT: renal replacement therapy.

Supplementary material 5

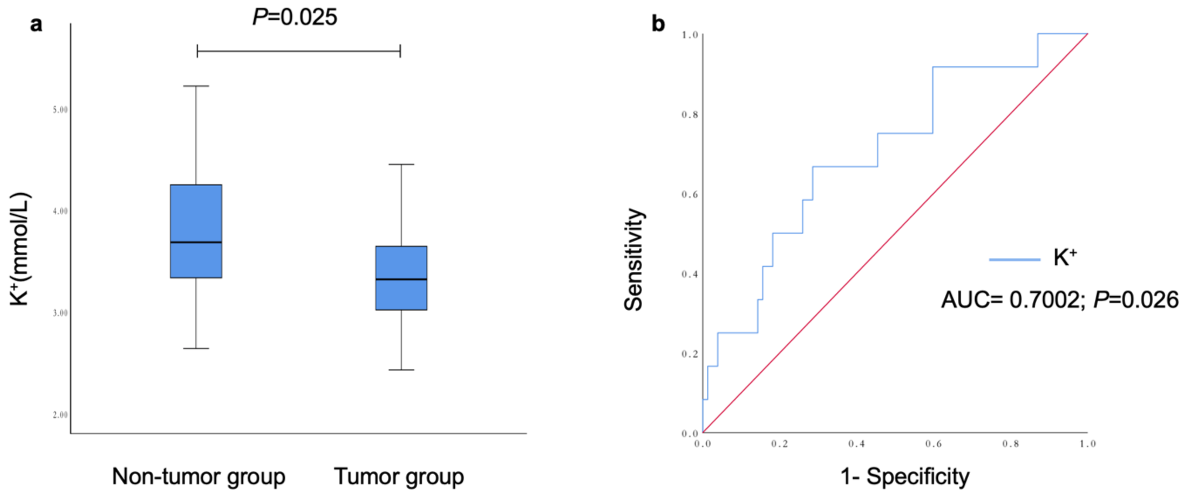

Fig.1 Visualizing the relationship between serum K^+ and tumor

(a) the difference of serum K^+ between tumor group and non-tumor group; (b) the serum K^+ model for tumor outcome using ROC curves.

Abbreviations: AAV: ANCA-associated vasculitis; ROC: receiver operating characteristic; AUC: Area Under ROC Curve.

Supplementary material 6

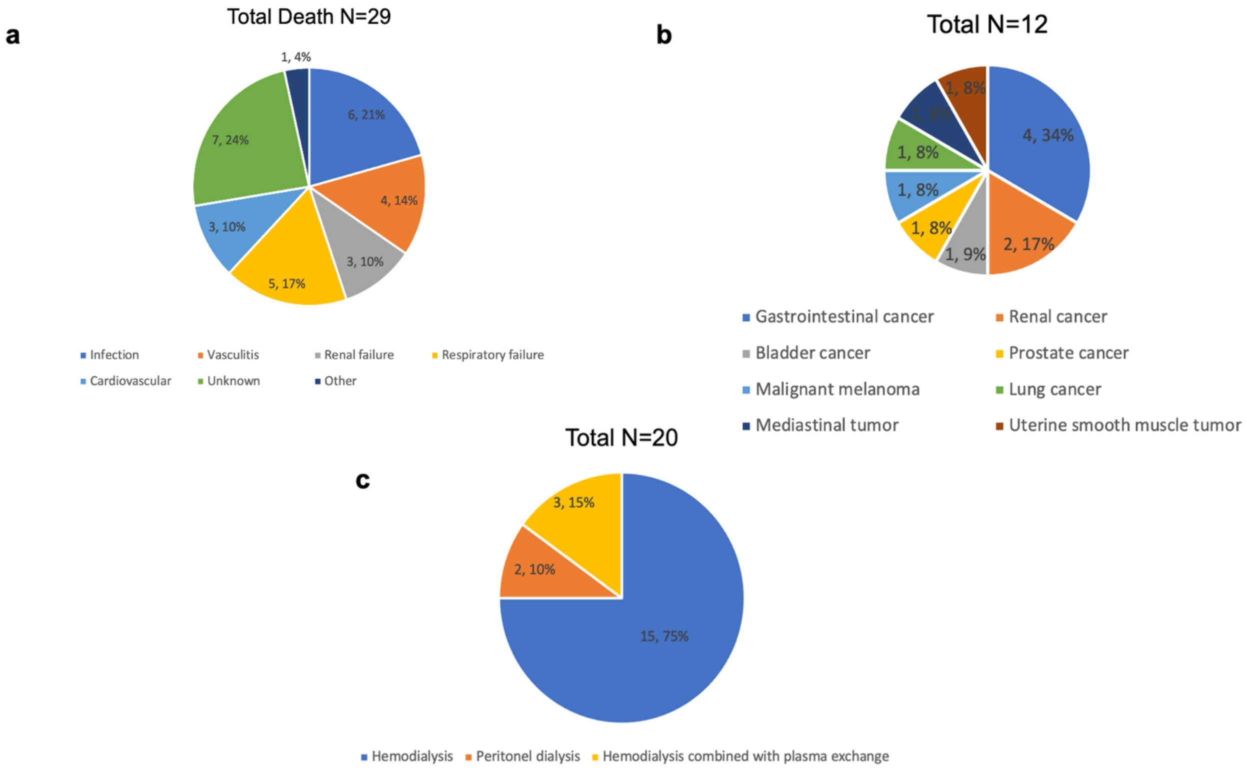

Fig.2 Main causes and types of high-risk events during the follow-up period

(a) The main cause of death in AAV patients; (b) The tumor type of AAV patients; (c) The renal replacement therapy type of AAV patients.

Abbreviations: AAV: ANCA-associated vasculitis.

Supplementary material 7

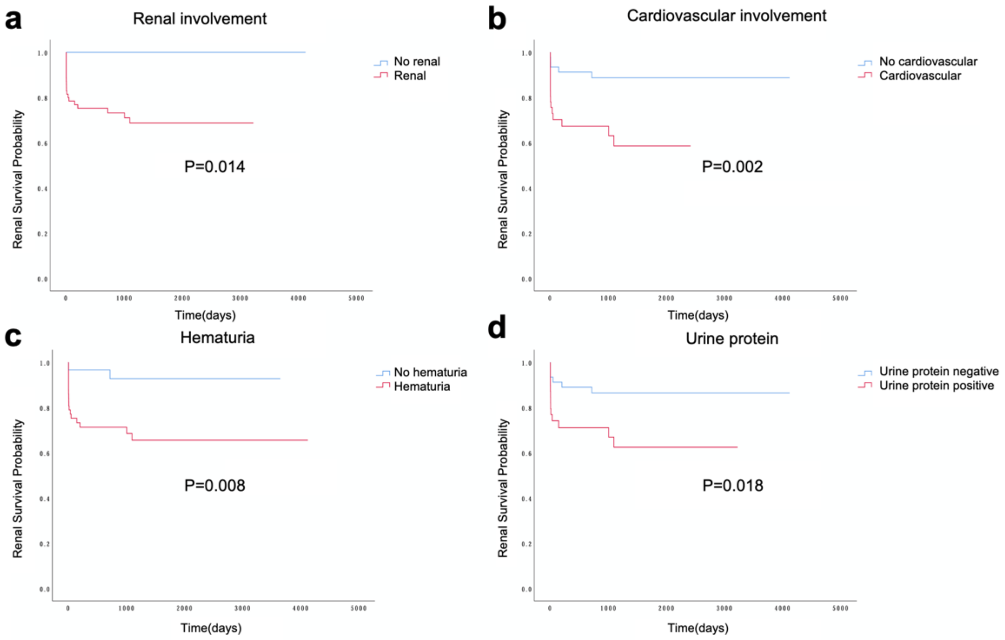

Fig.3 Comparison of renal survival rates between different subgroups in AAV

Renal survival by Renal involvement (a), Cardiovascular (b), Hematuria (c), Urine protein (d) at diagnosis; p-values obtained with the log-rank analysis.

Abbreviations: AAV: ANCA-associated vasculitis.

Supplementary material 8

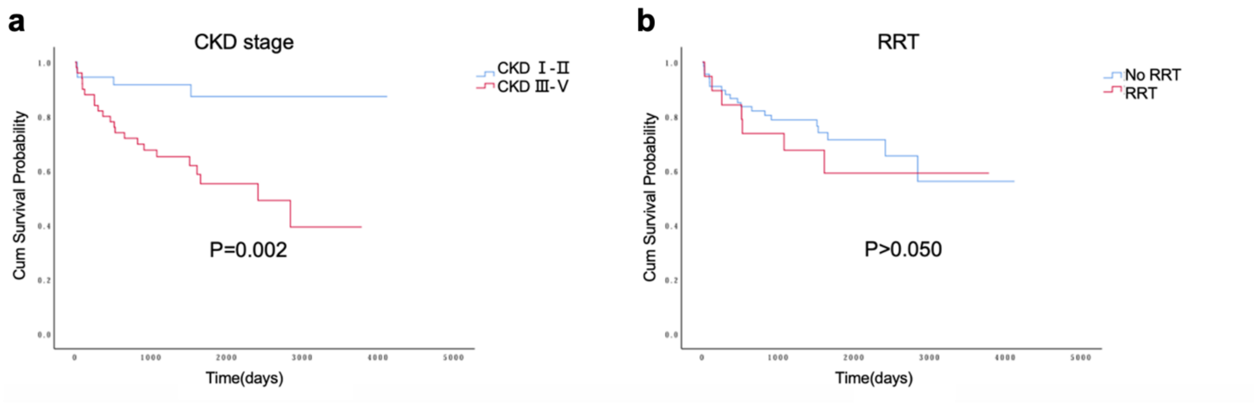

Fig.4 Comparison of cum survival rates between different subgroups in AAV

Cum survival of AAV patients by CKD stage (a), and RRT (b) status; p-values obtained with the log-rank analysis.

Abbreviations: AAV: ANCA-associated vasculitis; CKD: chronic kidney disease; RRT: renal replacement therapy.

Supplementary material 9

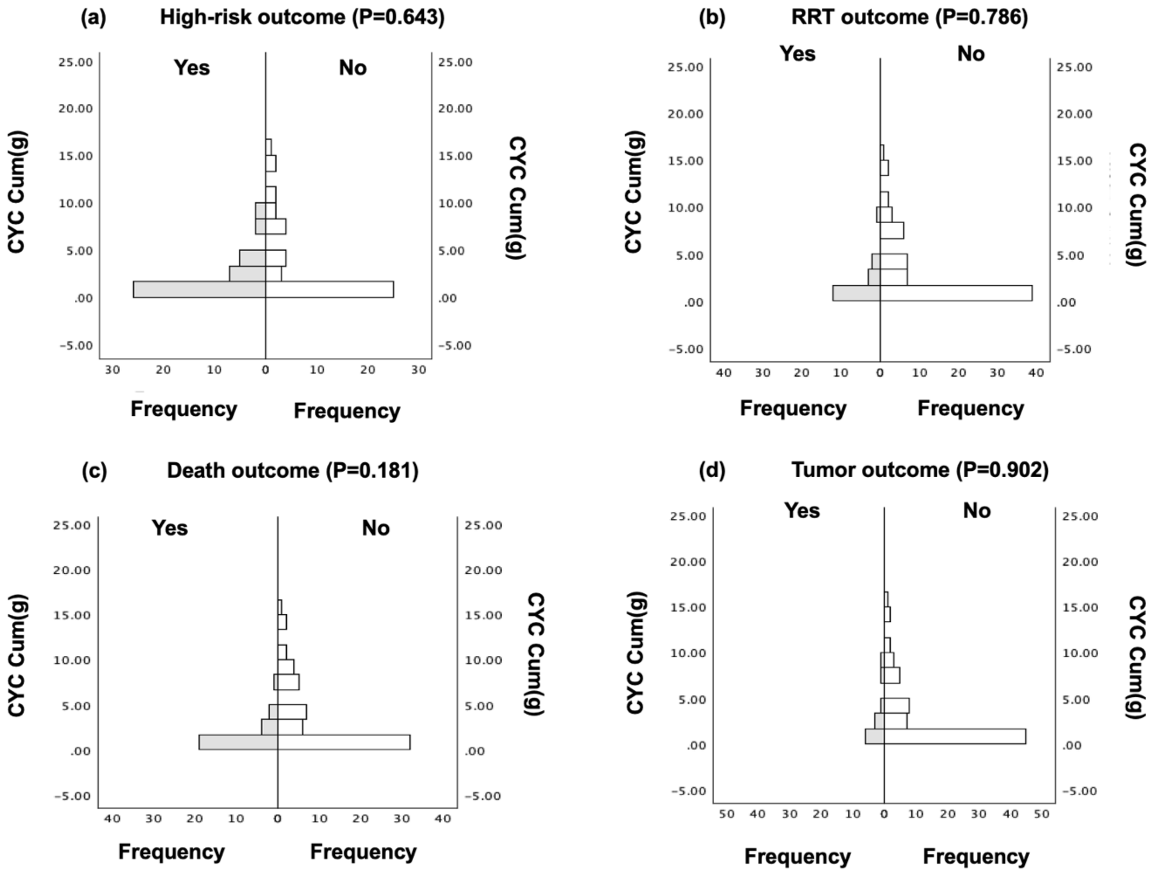

Fig.5 Correlation between cyclophosphamide accumulation and different outcomes

Cumulative dose use of CYC in AAV patients by high-risk outcome (a), RRT outcome (b), death outcome (c), and oncologic outcome (d); p-values obtained with the Mann-Whitney U test.

Abbreviations: AAV: ANCA-associated vasculitis; CYC: cyclophosphamide; Cum: cumulation; RRT: renal replacement therapy.

Supplementary material 10

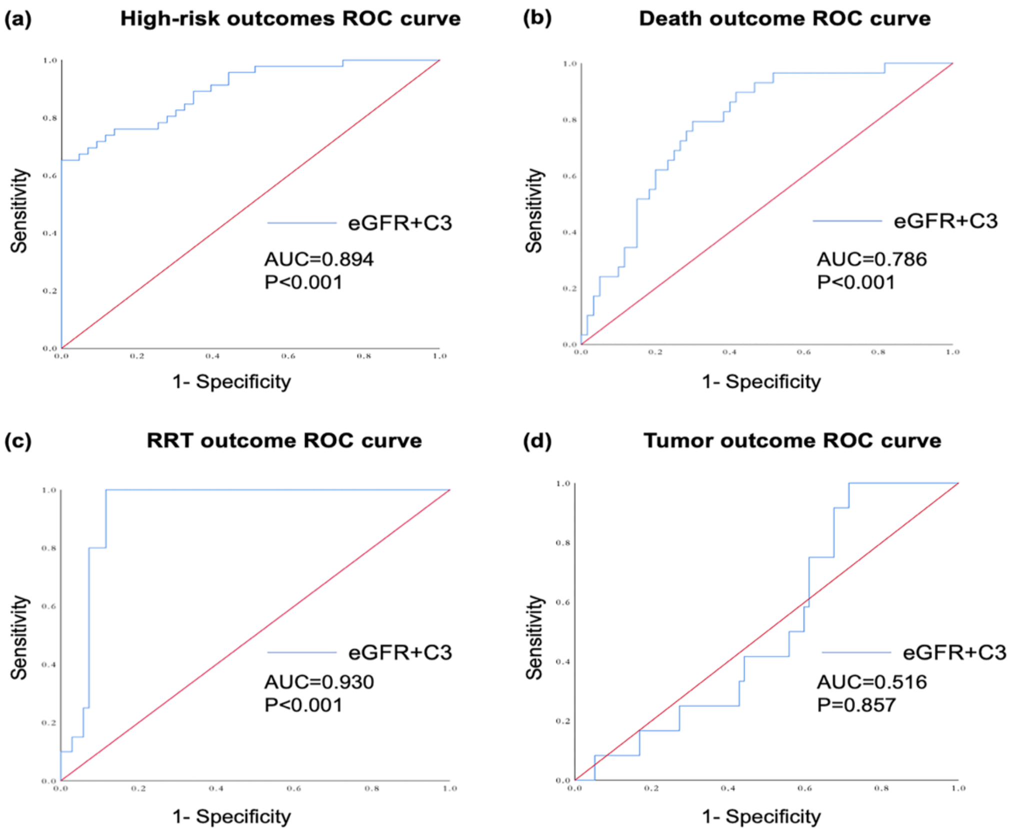

Fig.A1 ROC curves for different outcomes

Supplementary material 11

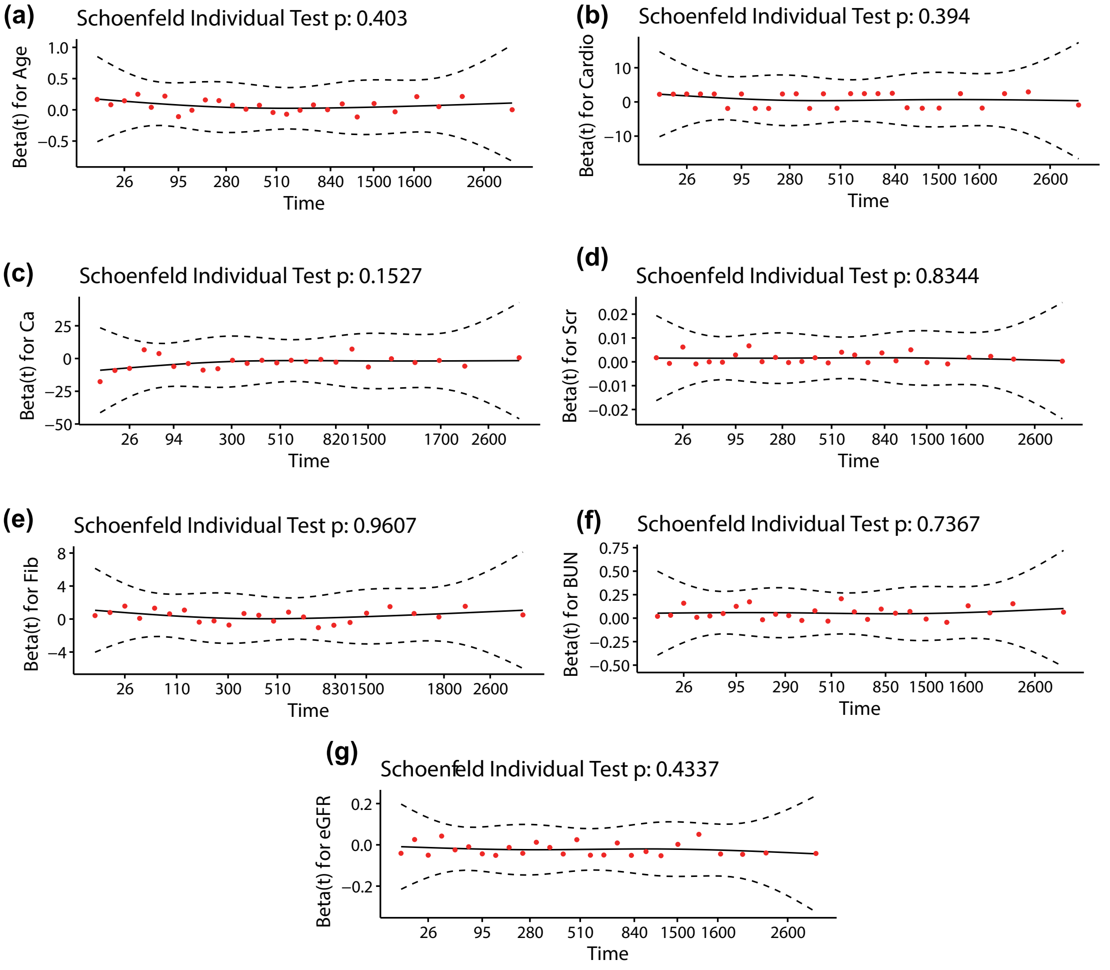

Fig.A2 A normalized correlation image of Schoenfeld residuals with respect to time for each covariate in death Cox regression model

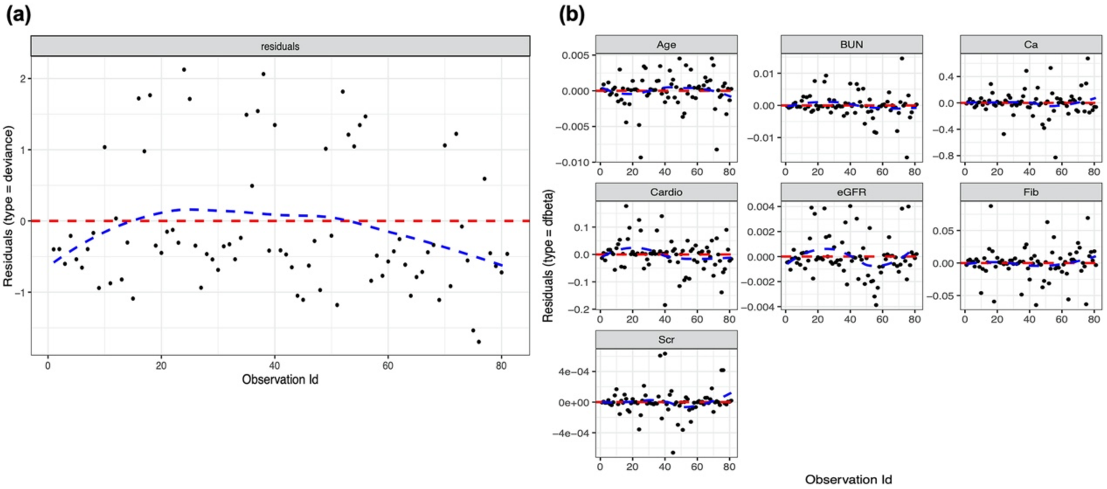

Fig.A3 The visualization of DFbeta values and Deviance residuals checking for outliers in death Cox regression model

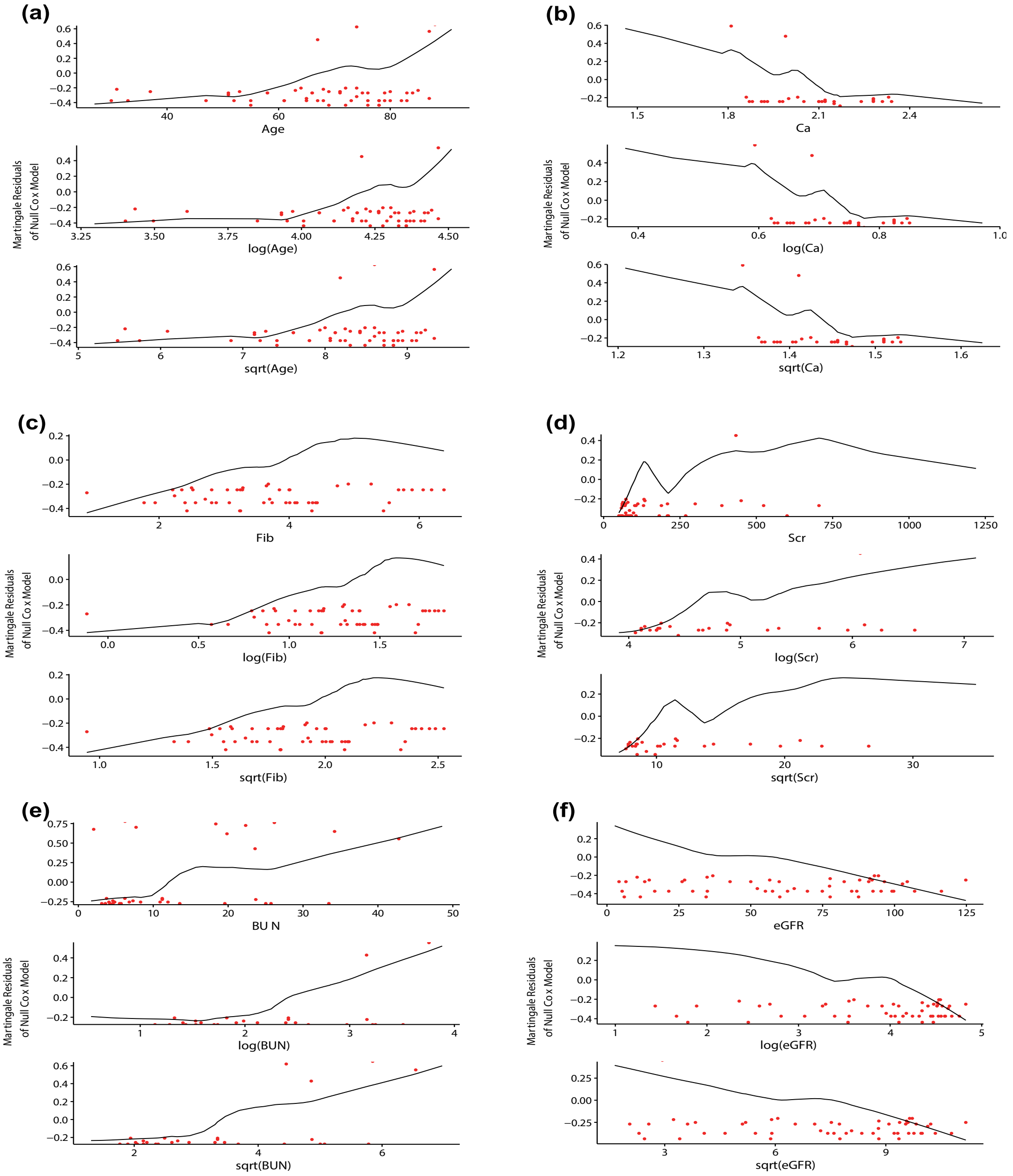

Fig.A4 The nonlinearity of the relationship between log hazard and continuous covariates in death Cox regression model

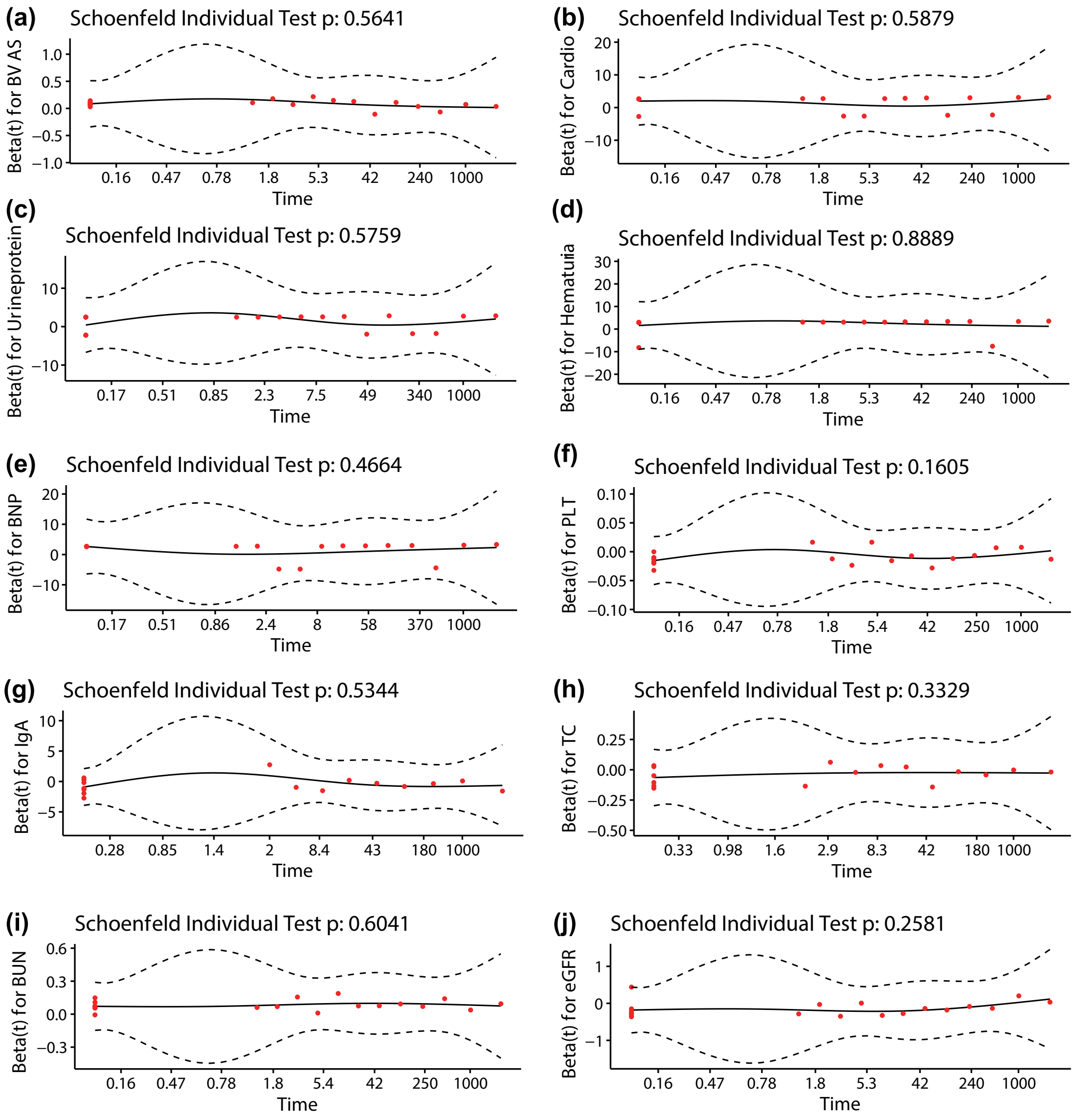

Fig.A5 A normalized correlation image of Schoenfeld residuals with respect to time for each covariate in renal Cox regression model

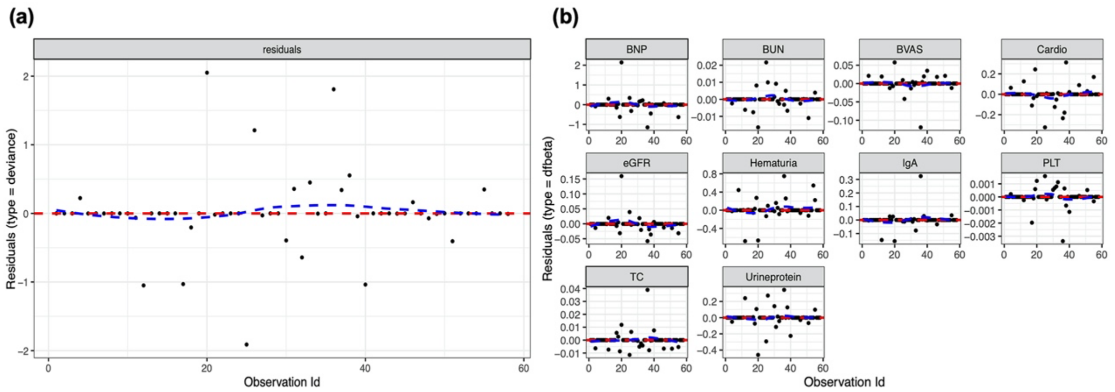

Fig.A6 The visualization of DFbeta values and Deviance residuals checking for outliers in renal Cox regression model

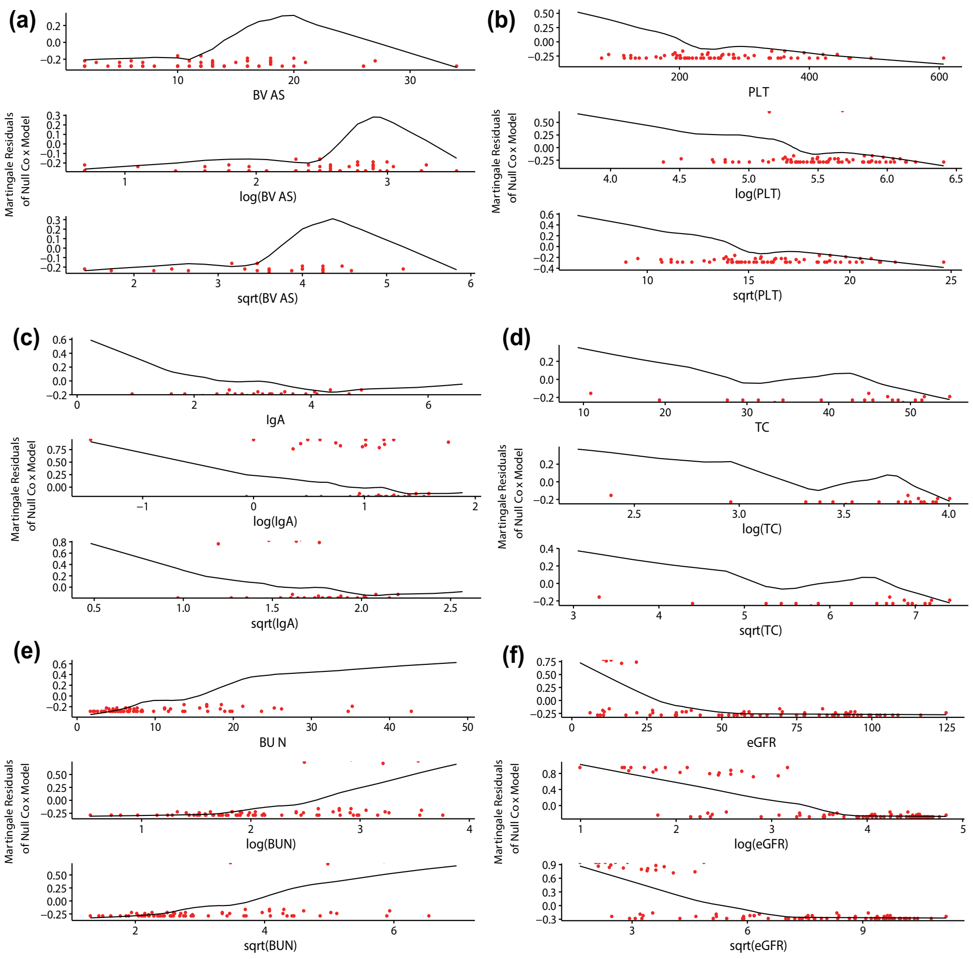

Fig.A7 The nonlinearity of the relationship between log hazard and continuous covariates in renal Cox regression model
